# Vitamin D status in children with mild, moderate, or severe confirmed COVID-19: systematic-review and meta-analysis

**DOI:** 10.3389/fped.2025.1436633

**Published:** 2025-05-13

**Authors:** Tahoora Mousavi, Mahmood Moosazadeh

**Affiliations:** ^1^ Molecular and Cell Biology Research Center, Hemoglobinopathy Institute, Faculty of Medicine, Mazandaran University of Medical Sciences, Sari, Iran; ^2^ Gastrointestinal Cancer Research Center, Non-Communicable Diseases Institute, Mazandaran University of Medical Sciences, Sari, Iran

**Keywords:** vitamin D, children, COVID-19, SARS coV-2, pneumonia

## Abstract

**Background:**

Vitamin D acts as a pro-hormone with a wide range of beneficial effects. It is reported that vitamin D deficiency is a risk factor for COVID-19 severity in children. In the present study, we decided to assess 25 hydroxy (OH) vitamin D status in children with mild, moderate, or severe confirmed COVID-19 and also compare them with those of a healthy control group using existing data.

**Methods:**

Relevant studies were extracted using online international databases including Scopus, Science Direct, PubMed, Web of Science, ProQuest, and Google Scholar search engine between Jan 2019 and 2024. The quality of all papers is determined by the NOS checklist. Heterogeneity between the results of primary studies was evaluated with the I-square index. Egger's test, funnel plot, and sensitivity analysis were applied. The statistical analysis was done using Stata version 17.

**Results:**

In 12 documents, the status of vitamin D was examined between case and control groups. By combining the results of these studies using random effect model, the standardized mean difference (SMD) vitamin D level in the COVID-19 children compared to the control group was estimated to be −0.88 (98% CI: −1.24, −0.51), which was statistically significant. In the present study, the odd ratio of vitamin D deficiency and vitamin D disorder (insufficiency and deficiency) in children with moderate COVID-19 compared to asymptomatic children with COVID-19 were estimated to be 3.58 (1.10, 11.63) and 2.52 (0.99, 6.41) respectively which was higher than in asymptomatic children with COVID-19. In addition, vitamin D deficiency and vitamin D disorder in children with moderate COVID-19 compared to the children with mild COVID-19 were estimated to be 2.12 (0.90, 4.98) and 1.82 (0.78, 4.22) respectively, which was higher than in children with mild COVID-19. Also, vitamin D deficiency and vitamin D disorder in children with mild COVID-19 compared to asymptomatic children with COVID-19 were estimated to be 2.02 (0.60, 6.78) and 1.64 (0.53, 5.07) respectively, which was higher than in asymptomatic children.

**Conclusions:**

Combining the results of these studies, the effect size of the relationship between vitamin D and COVID-19 in children is significant. During the COVID-19 pandemic (except for the Omicron peak), children were less affected by the severity of COVID-19. The standardized mean difference (SMD) vitamin D level in children with COVID-19 was significantly 0.88 units lower than the control group. Also, the odds ratio of moderate COVID-19 in children with vitamin D deficiency was significantly 3.58 times higher than in asymptomatic children with COVID-19.

## 1 Introduction

In December 2019, a new coronavirus (CoV) infection started with pneumonia epidemics in Wuhan, Hubei, and China, and spread throughout the world, resulting in the COVID-19 pandemic (1, 2). COVID-19 infection has been reported in different age groups and results in high mortality in patients (3). The first confirmed cases among children were reported from January 20 to February 6, 2020, in children (≤18 years) (4). In children, COVID-19 usually occurs with milder symptoms; however, cases with more severe disease have been reported (5). The most common symptoms of acute respiratory infection in the pediatric age group are fever, watery diarrhea, frequent runny noses, fever, cough, nausea and vomiting (6).

It has been reported that hyper-inﬂammation increases the mortality risk in severe COVID-19 patients (7) and vitamin D has a protective effect against acute respiratory interstitial pneumonia, and influenza A infections (8). Vitamin D acts as a pro-hormone (prototypical secosteroid) with a wide range of beneficial effects, including immune-modulator, anti-inflammatory, anti-fibrotic, epithelial integrity protective properties and antioxidant effects. Vitamin D upregulates IL-4 in Th2 cells and inhibits inflammatory cytokine expression and Th1 cell proliferation. It is suggested that vitamin D deficiency is associated with overexpression of Th1 cytokines (9, 10). The finding showed that vitamin D produces cathelicidin (an endogenous antimicrobial peptide), reduces the synthesis of dipeptidyl peptidase-4 receptor (DPP-4/CD26), and plays a protective role with antibacterial, antiviral, and antioxidant properties (11).

Angiotensin-converting enzyme II (ACE2) is the cell receptor for SARS-CoV-2 and is not as robust in children as it is in adults. 1,25(OH)2D3, The active form of vitamin D, can induce ACE2 expression which plays a role in protecting against severe infection and its level is associated with asymptomatic or mild-to-moderate symptoms of COVID-19 (3).

It is reported that vitamin D deficiency, an important public health problem, is a risk factor for COVID-19 severity in children. It has been reported that vitamin D supplementation is associated with reduced mortality risk, length of hospital stay, and ICU admission rates in COVID-19 patients (12). Different cutoffs of vitamin D exist. Following recent consensus, deficiency was defined as 25(OH)D concentration of ≤50 nmol/L (20 ng/ml) and vitamin D insufficiency or deficiency are common findings in children (13).

In the present study, we decided to assess 25 hydroxy (OH) vitamin D status in children with mild, moderate, or severe confirmed COVID-19 and also compare them with those of a healthy control group using existing data.

## 2 Material and methods

This study was designed and conducted based on Preferred Reporting Items for Systematic Reviews and Meta-Analyses (PRISMA) guidelines, but its protocol was not registered.

The primary outcome was the assessment of 25-Hydroxy vitamin D status in case (COVID-19 patients) and control (healthy) groups. The secondary outcome was the evaluation of 25-Hydroxy vitamin D in patients with asymptomatic, mild, moderate, and severe COVID-19.

### 2.1 Search strategy

In the present study, two researchers (M.M and T.M) conducted the literature search independently. The published articles were collected via a systematic search of the literature databases such as Scopus, Science Direct, Pubmed, Web of Science**,** Proquest, and Google Scholar search engine between January 2019 and 2024.

Our search terms included “ vitamin D”, “vitamin D Deficiencies”, “25-Hydroxyvitamin D2”, “COVID-19”, “SARS-COV-2”, “Children”, and “Pediatric” by the combination of “OR”, and “AND” Boolean Operators in the Title/Abstract/Keywords field (Table 1). In addition, the article references were screened to find additional related studies and increase search sensitivity. Finally, all collected references are entered into reference management software (EndNote). A reference list of all related studies was also reviewed for any other related publications. One of the team's researchers randomly evaluated the search results and reported that no relevant study was ignored. The search was restricted to original Articles/Abstracts published in the English language that reported the association between vitamin D status and COVID-19 infection in children.

**Table 1:** Search strategy.

Search study for vitamin D status in children with COVID-19
(″ Vitamin D ″ [MeSH Terms]) AND (″ COVID-19″ [ MeSH Terms]) OR (″ Vitamin D ″ [MeSH Terms]) AND (″ Children″ [ MeSH Terms]) OR (″ Vitamin D ″ [MeSH Terms]) AND (″ Pediatric″ [ MeSH Terms]) OR (″ Vitamin D ″ [ MeSH Terms ]) AND (″ SARS-COV-2″[ MeSH Terms ]) OR (″ Vitamin D Deficiencies ″ [MeSH Terms]) AND (″ COVID-19″ [ MeSH Terms]) OR (″ Vitamin D Deficiencies ″ [MeSH Terms]) AND (″ Children″ [ MeSH Terms]) OR (″ Vitamin D Deficiencies ″ [MeSH Terms]) AND (″ Pediatric″ [ MeSH Terms]) OR (″ Vitamin D Deficiencies ″ [ MeSH Terms ]) AND (″ SARS-COV-2″[ MeSH Terms ]) OR (″ 25-Hydroxyvitamin D2 ″ [MeSH Terms]) AND (″ COVID-19″ [ MeSH Terms]) OR (″ 25-Hydroxyvitamin D2 ″ [ MeSH Terms ]) AND (″ SARS-COV-2″[ MeSH Terms ]) OR (″ 25-Hydroxyvitamin D2 ″ [MeSH Terms]) AND (″ Children″ [ MeSH Terms]) OR (″ 25-Hydroxyvitamin D2 ″ [MeSH Terms]) AND (″ Pediatric″ [ MeSH Terms])

All these steps were done by two authors (M.M and TM) and any disagreements with article selection were resolved through discussion, and a third author (A.N) was available to resolve the disagreement. In addition, the article references were screened to find additional related studies and increase search sensitivity. Finally, all collected references are entered into reference management software (EndNote).

### 2.2 Inclusion criteria

**“**P” signifies children patients (1 month-18 years) with COVID-19; “I (E)” means 25-Hydroxy vitamin D. “C” Healthy groups (control). “O” means the assessment of 25-Hydroxy vitamin D in case (COVID-19 patients) and control (healthy) groups and the assessment of 25-Hydroxy vitamin D in asymptomatic, moderate, mild, and severe groups.

All cohort or case-control studies were entered into this meta-analysis for the assessment of 25-Hydroxy vitamin D in the case and control groups.

### 2.3 Exclusion criteria

1. Studies that scored less than 5 based on the quality assessment checklist were excluded from the meta-analysis.
2. Duplicate publications, reviews, animal research, case reports, *in vitro*, and in-silico studies were removed from the meta-analysis.
3. Research of adult groups (14).
4. Research of specific groups (15).
5. Studies that did not report the disease severity (mild/moderate or severe) (16–20).
6. Studies were not case-control (16, 21).

### 2.4 Study selection

**Figure 1:**
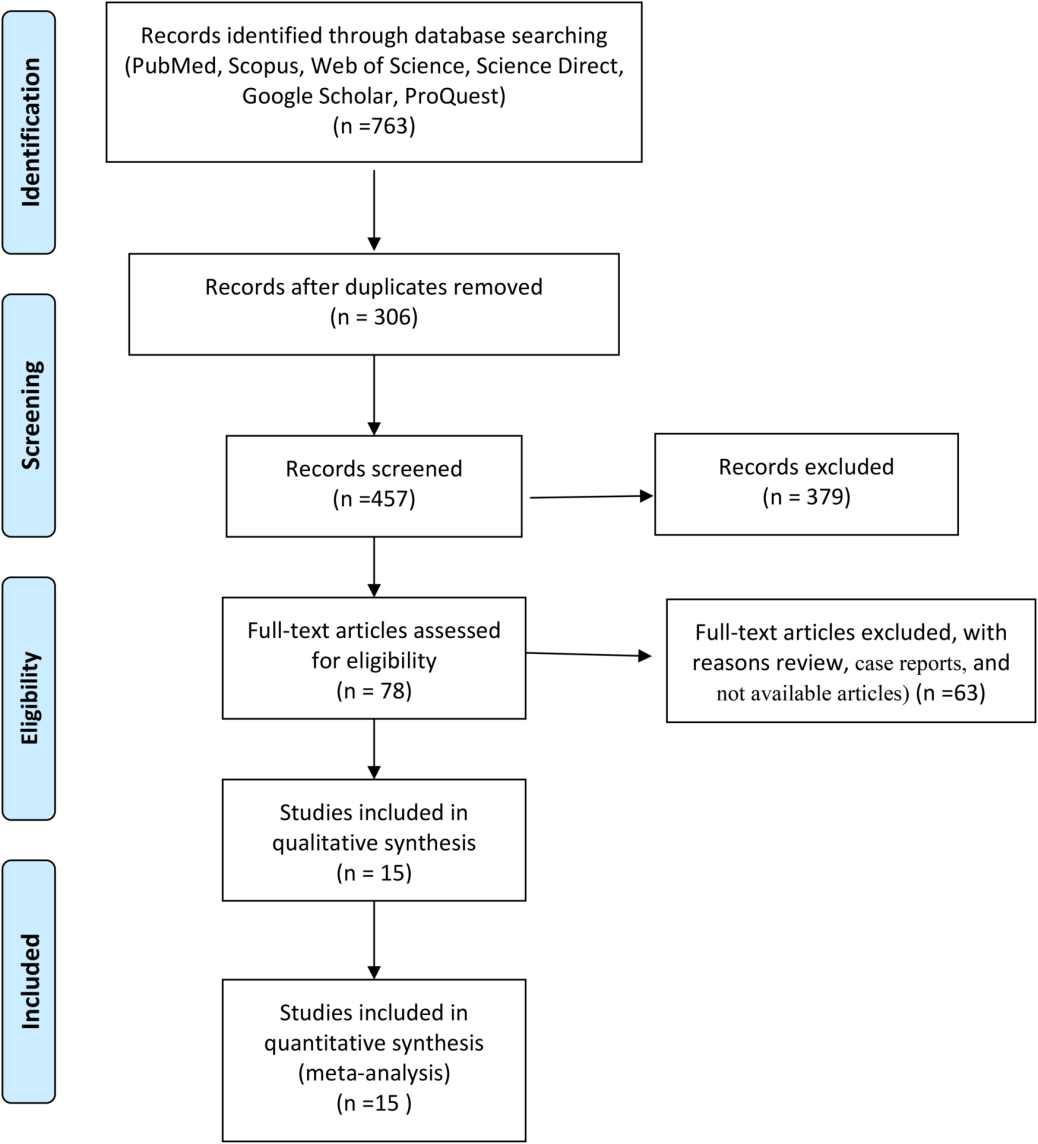
Flowchart of included studies.

First, the full text or summary of articles, documents, and reports is extracted from different databases. After removing duplicates, the irrelevant articles were excluded from this study. Then, full-text articles were assessed for eligibility and next, review studies were removed from this meta-analysis. Finally, data was extracted from full-text articles based on inclusion and exclusion criteria (Figure 1).

### 2.5 Data extraction

For data extraction, two researchers independently screened the studies based on article title, first author's name, year of study, place of study, type of study, the total number of cases and controls, mean (SD) of vitamin D in the case group, mean (SD) of vitamin D in the control group, the median of vitamin D in the case group, the median of vitamin D in the control group, number of asymptomatic, mild, moderate and severe patients with normal vitamin D, number of asymptomatic, mild, moderate and severe patients with vitamin D insufficiency, and number of asymptomatic, mild, moderate and severe patients with vitamin D deficiency.

### 2.6 Quality evaluation

Considering all the prospective cohort studies entered in our meta-analysis, the Newcastle–Ottawa scale (NOS) checklist was applied to assess the quality of studies. Based on this checklist, nine questions assigning a score and covering the type of study, sample size, study objectives, study population, sampling method, data analysis method, presentation of findings in an appropriate way, and presentation of results based on objectives were designed. This checklist has three parts: selection, comparability, and exposure (range 0–9). Only the studies that scored at least 5 points were included in the study. Maximum criterion scores (2); maximum comparability scores (2); and maximum exposure has 3 scores (22, 23).

### 2.7 Analysis

In the present study, Stata version 17 (Stata Corp, College Station, TX, USA) was applied for data analysis. In this study, to estimate the odd ratio, a two-by-two table was designed for each of the primary studies. Data were combined based on the random effect model and DerSimonian–Laird method. The odds ratio with a 95% confidence interval is presented in a Forest plot.

The standardized mean difference (SMD) vitamin D level, the number of samples, the mean and the standard deviation of vitamin D were extracted from primary studies. Using the random effect model and DerSimonian–Laird method, the SMD of vitamin D between the case and control groups was estimated with a 95% confidence interval. The criterion for judging the significant differences between SMD of vitamin D does not include the zero between the upper and lower confidence interval of the SMD.

For assessing the heterogeneity index between studies, the Cochran (*Q*) and I2 tests were used. Egger's test and funnel plot were applied to assess publication bias and sensitivity analysis was performed to determine the influence of individual studies on the overall estimate.

## 3 Results

### 3.1 Comparison of the vitamin D between case and control groups

**Figure 2:**
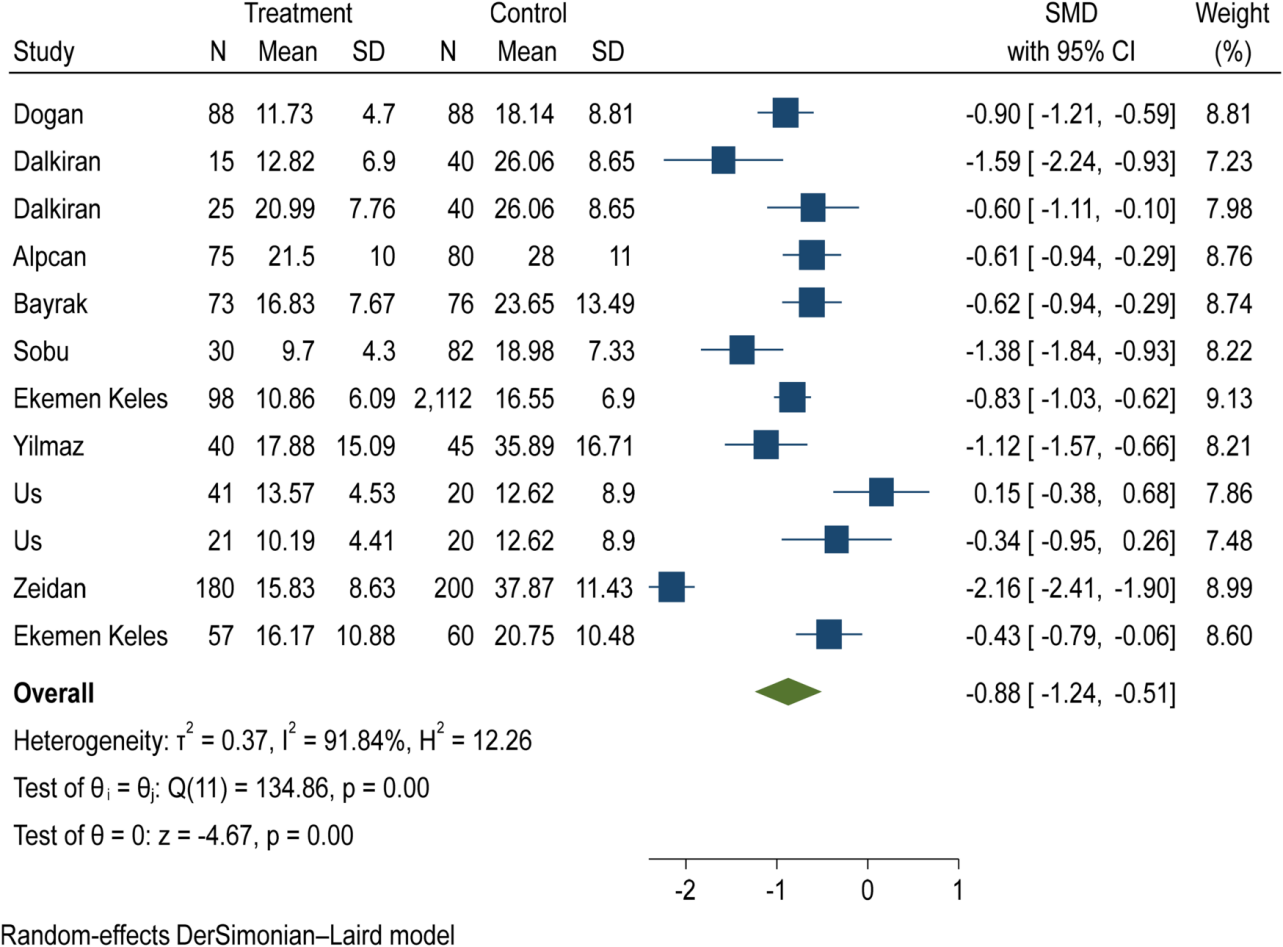
The pooled estimates of SMD of vitamin D between the case and control groups according to primary studies and overall estimate with a 95% confidence interval.

The average of vitamin D level in the case and control group was examined in 12 evidence (Table 2). In 11 studies, the SMD of vitamin D in the case group was lower than the control group, while in 10 studies, the observed differences were statistically significant. According to the results of heterogeneity indices (*I*-square: 91.84%, *Q*: 134.86, *P*-value < 0.001), the heterogeneity between primary studies was high. Using DerSimonian–Laird method and a random effect model, the results of 12 primary studies were combined, and the standardized mean difference (SMD) vitamin D level in the COVID-19 children compared to the control group was estimated to be −0.88 (98% CI: −1.24, −0.51), which was statistically significant (Figure 2).

**Table 2:** Characteristics of case-control studies.

Author	Year	Country	Sample size cases	Age	Mean cases	SD cases	Sample size controls	Mean controls	SD controls
Dogan et al. (48)	2022	Turkey	88	1–18 years	11.73	4.7	88	18.14	8.81
Dalkiran and Sevcan (49)	2023	Turkey	15	1 month–18 years	12.82	6.9	40	26.06	8.65
Dalkiran and Sevcan (49)	2023	Turkey	25	1 month–18 years	20.99	7.76	40	26.06	8.65
Alpcan et al. (10)	2021	Turkey	75	1–18 years	21.5	10	80	28	11
Bayrak et al. (3)	2023	Turkey	73	1 month–18 years	16.83	7.67	76	23.65	13.49
Söbü et al. (50)	2021	Turkey	30	1–17.6 years	9.7	4.3	82	18.98	7.33
Ekemen Keles et al. (24)	2023	Turkey	98	1 month–18 years	10.86	6.09	2112	16.55	6.9
Yılmaz and Sen (26)	2020	Turkey	40	1 month–18 years	17.88	15.09	45	35.89	16.71
Us et al. (51)	2021	Turkey	41	0–18 years	13.57	4.53	20	12.62	8.9
Us et al. (51)	2021	Turkey	21	0–18 years	10.19	4.41	20	12.62	8.9
Zeidan et al. (52)	2023	Egypt	180	<19	15.83	8.63	200	37.87	11.43
Ekemen Keles et al. (28)	2022	Turkey	57	1 month–18 years	16.17	10.88	60	20.75	10.48

**Figure 3:**
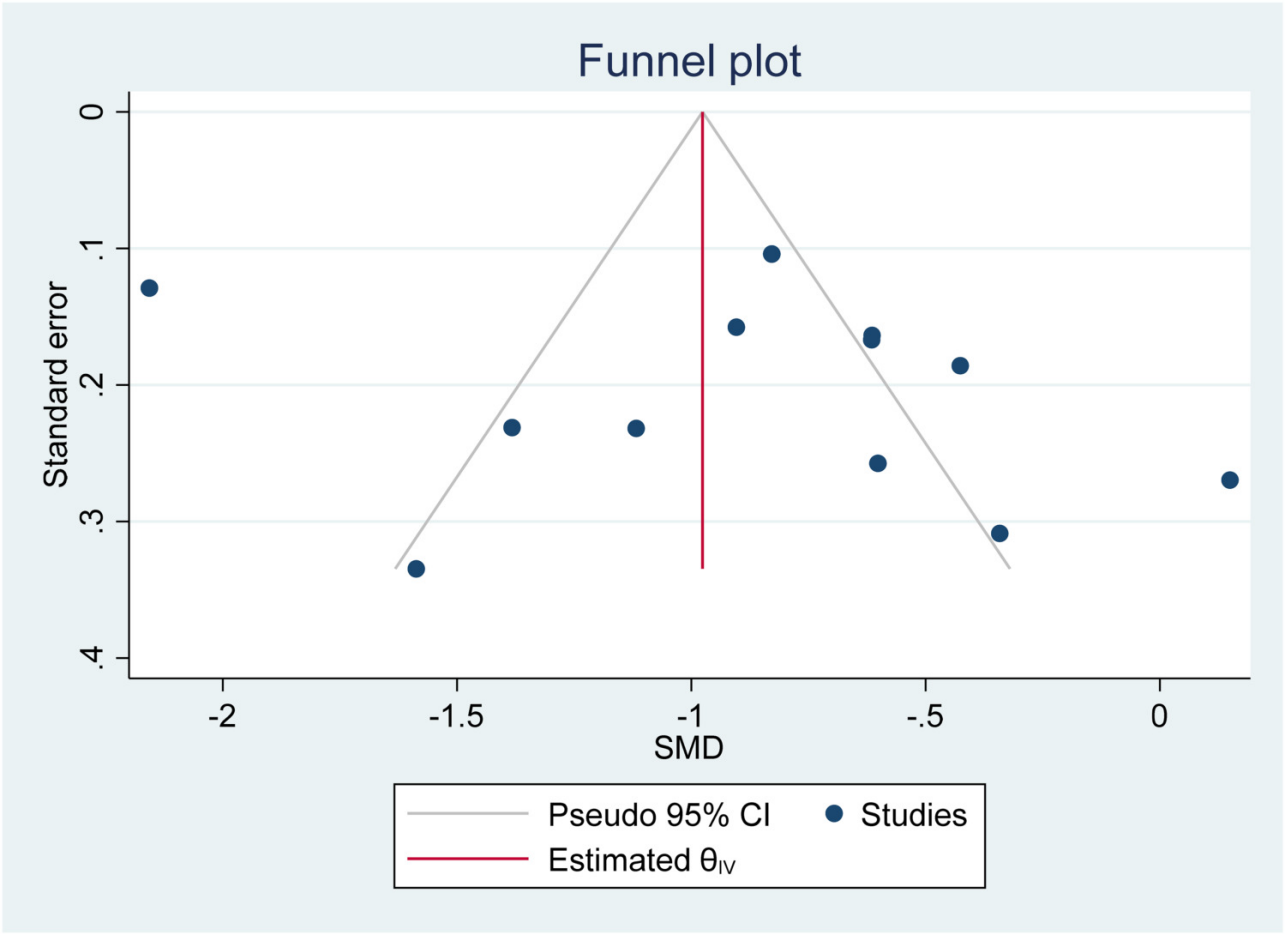
Investigate the publication bias of primary studies in estimating the SMD of vitamin D between the case and control group.

**Figure 4:**
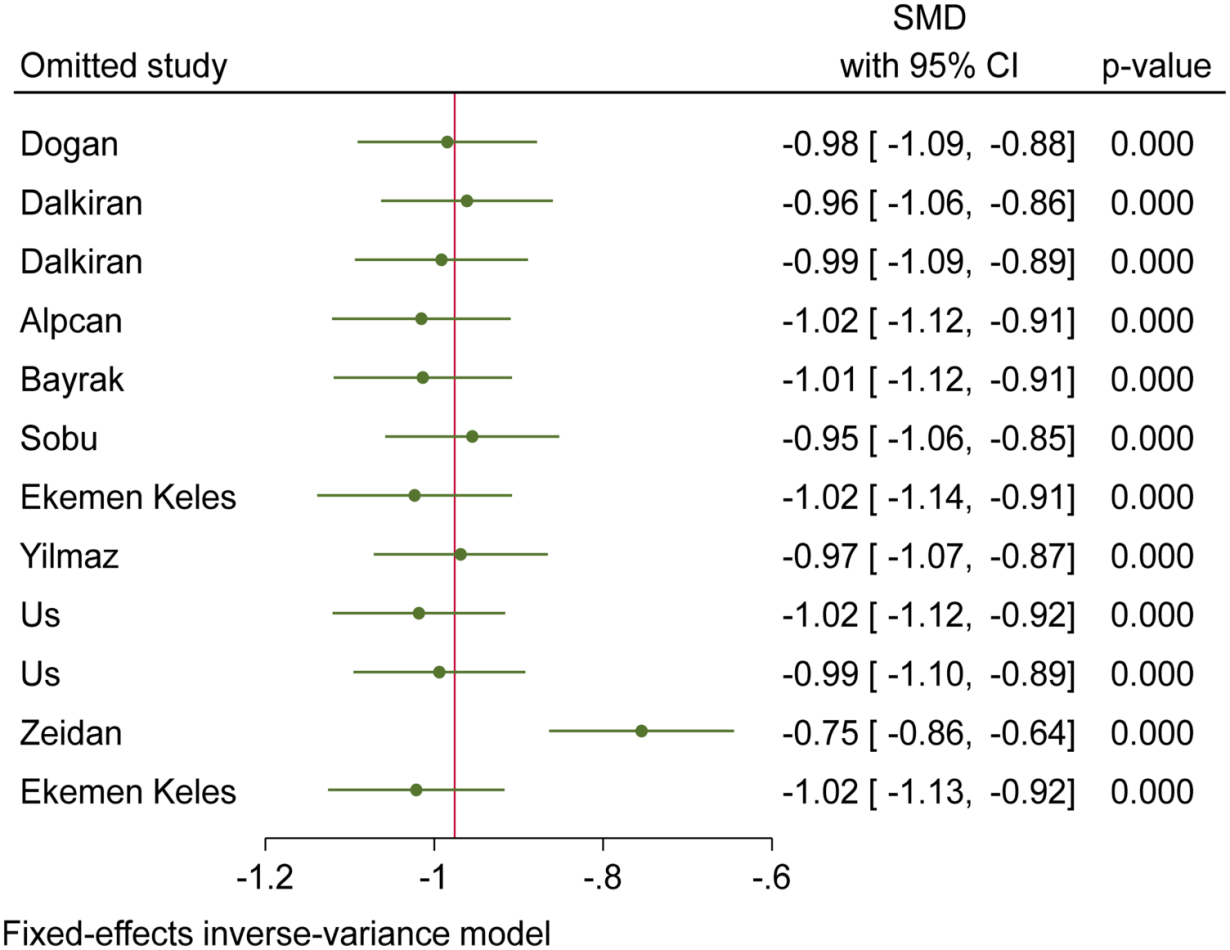
Sensitivity analysis chart to investigate the effect of each of the primary studies on the overall estimation of SMD of vitamin D between the case and control group.

Based on the funnel plot diagram (Figure 3) and the results of Egger's test (*β* = 1.74, *P*-value: 0.551), it can be stated that there is no publication bias to estimate the SMD of vitamin D between the case and the control group. Also, the results of the sensitivity analysis show that there are no differences between the effect of each primary study on the overall estimate of SMD of vitamin D between the case and control groups (Figure 4).

### 3.2 Vitamin D and COVID-19 severity

**Figure 5:**
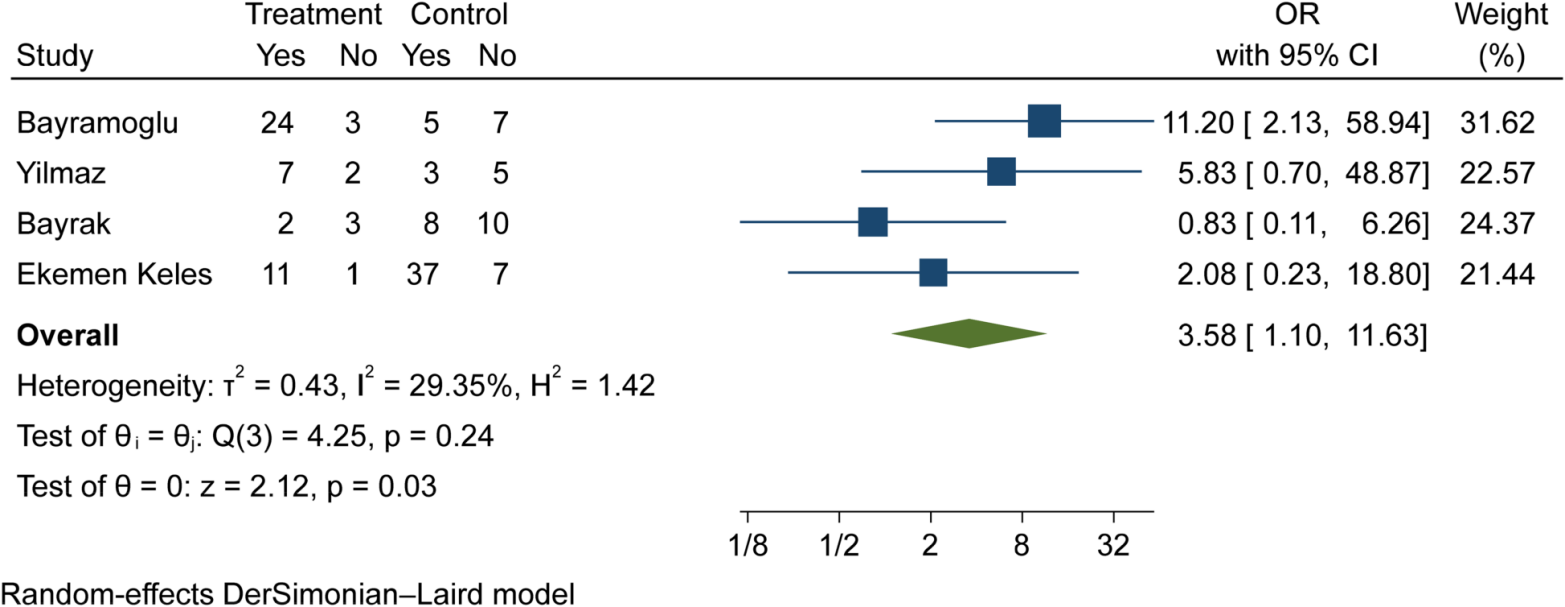
Comparison of vitamin D deficiency among children with moderate COVID-19 compared to the asymptomatic children with COVID-19.

Vitamin D (deficiency, insufficiency, and normal) was evaluated in COVID-19 children (Tables 3–6). The comparison of vitamin D deficiency between children with moderate COVID-19 compared to asymptomatic children with COVID-19 was investigated in four primary studies. In three of these studies, the odds ratio for moderate COVID-19 in children with vitamin D deficiency [ranged from 0.83 in Ekemen Keles (24) to 11.2 in Bayramoglu's study (25)] was higher than in asymptomatic children with COVID-19. Differences were statistically significant only in the study of Bayramoglu et al. (25). By combining the results of these studies, the odds ratio for contracting vitamin D deficiency among children with moderate COVID-19 (OR: 3.58, 95% CI: 1.10, 11.63) was 3.58 times that of asymptomatic children; which is statistically significant. It should be noted that the heterogeneity between the studies was not significant (*I*-square: 29.35%, *Q*: 4.25, *P*-value: 0.24) (Figure 5).

**Table 3:** Vitamin D status in asymptomatic children with COVID-19 group.

Author	Year	Type study	Total	Asymptomatic number	Normal	Insufficiency	Deficiency	Insufficiency, deficiency
Bayramoğlu et al. (25)	2021	cohort	103	29	7	17	5	22
Yılmaz and Sen (26)	2020	Case-control	40	8	5	NA	3	3
Bayrak et al. (3)	2023	Case-control	73	18	10	NA	8	8
Ekemen Keles et al. (24)	2023	retrospective observational	98	44	7	NA	37	37

**Table 4:** Vitamin D status in mild group.

Author	Year	Type study	Total	Mild cases number	Normal	Insufficiency	Deficiency	Insufficiency, deficiency
Bayramoğlu et al. (25)	2021	Cohort	103	40	9	17	14	31
Karimian et al. (27)	2022	Cross-sectional	99	18	18	NA	NA	0
Yılmaz and Sen (26)	2020	Case-control	40	21	4	NA	17	17
Bayrak et al. (3)	2023	Case-control	73	50	36	NA	14	14
Ekemen Keles et al. (24)	2023	Retrospective observational	98	41	2	NA	39	39
Ekemen Keles et al. (28)	2022	Prospective	57	17	0	9	8	17

**Table 5:** Vitamin D status in moderate group.

Author	Year	Type study	Total	Moderate cases number	Normal	Insufficiency	Deficiency	Insufficiency, deficiency
Abdelrazic et al. (53)	2023	Cross-sectional	56	24	21	1	2	3
Bayramoğlu et al. (25)	2021	Cohort	103	34	3	7	24	31
Karimian et al. (27)	2022	Cross-sectional	99	60	49	NA	11	11
Yılmaz and Sen (26)	2020	Case-control	40	9	2	NA	7	7
Bayrak et al. (3)	2023	Case-control	73	5	3	NA	2	2
Ekemen Keles et al. (24)	2023	Retrospective observational	98	12	1	NA	11	11
Zengin et al. (54)	2022	Retrospective	34	20	0	6	14	20
Zeidan et al. (52)	2023	Prospective	180	134	66	NA	68	68
Ekemen Keles et al. (28)	2022	Prospective	57	22	0	15	7	22

**Table 6:** Vitamin D status in severe, and critical group.

Author	Year	Type study	Total	Severe, critical cases number	Normal	Insufficiency	Deficiency	Insufficiency, deficiency
Abdelrazic et al. (53)	2023	Cross-sectional	56	32	13	9	10	19
Karimian et al. (27)	2022	Cross-sectional	99	21	12	9	NA	9
Yılmaz and Sen (26)	2020	Case-control	40	2	0	NA	2	2
Bayrak et al. (3)	2023	Case-control	73	0	0	NA	0	0
Ekemen Keles et al. (24)	2023	Retrospective observational	98	1	NA	NA	1	1
Zengin et al. (54)	2022	Retrospective	34	14	0	1	13	14
Torpoco Rivera et al. (55)	2022	Retrospective	31	31	21	NA	10	10
Zeidan et al. (52)	2023	Prospective	180	46	20	NA	26	26
Ekemen Keles et al. (28)	2022	Prospective	57	18	0	14	4	18

**Figure 6:**
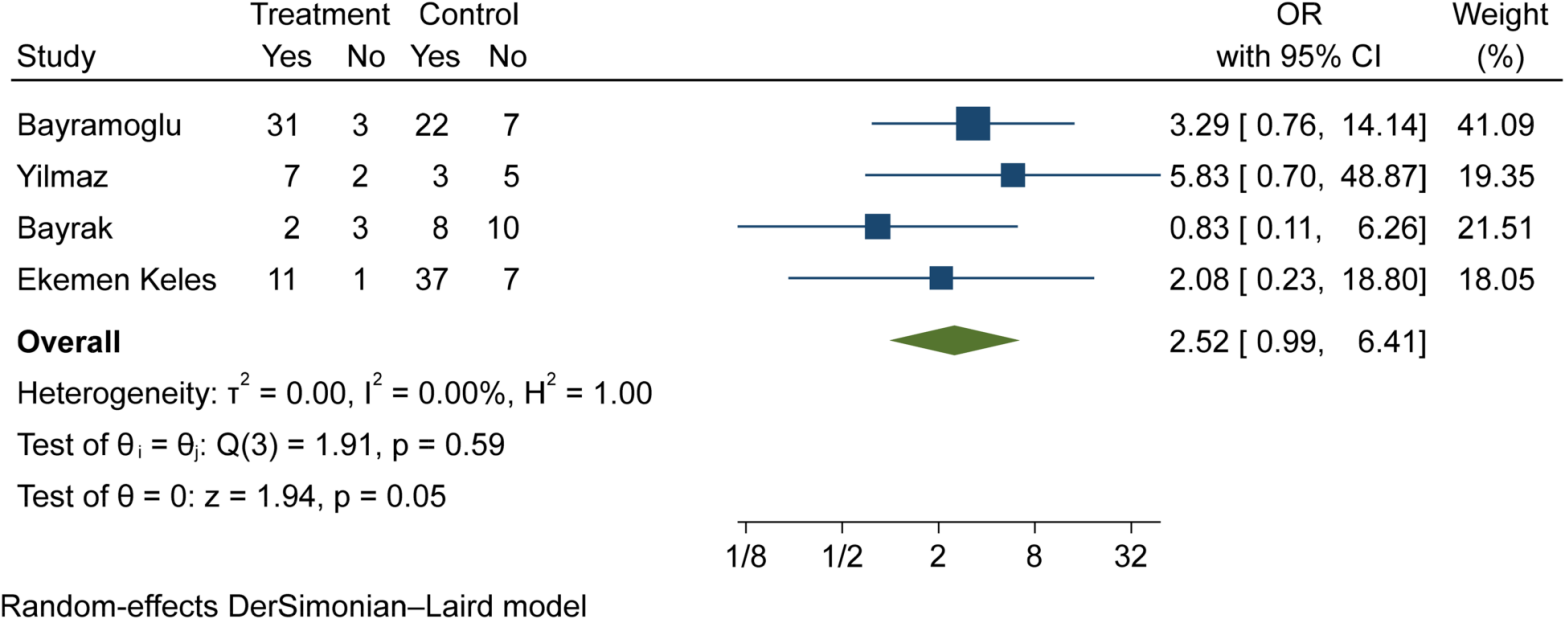
Comparison of vitamin D disorder (insufficiency and deficiency) among children with moderate COVID-19 compared to the asymptomatic children with COVID-19.

The comparison of vitamin D disorder in children with moderate COVID-19 compared to asymptomatic children with COVID-19 was investigated in four primary studies. In three of these studies, the odds ratio for moderate COVID-19 among children with vitamin D disorder was higher (from 0.83 in the Ekemen Keles study (24) to 5.83 in Yilmaz's study (26)) than in asymptomatic children with COVID-19. Despite the high effect size, statistically significant differences were not observed in the primary studies. By combining the results of these studies, the odds ratio of contracting moderate COVID-19 among children with vitamin D disorder (OR: 2.52, 95% CI: 0.99, 6.41) was 2.52 times that of asymptomatic children with COVID-19, which is not statistically significant. It should be noted that the heterogeneity between the studies was not significant (*I*-square: 0%, *Q*: 1.91, *P*-value: 0.59) (Figure 6).

**Figure 7:**
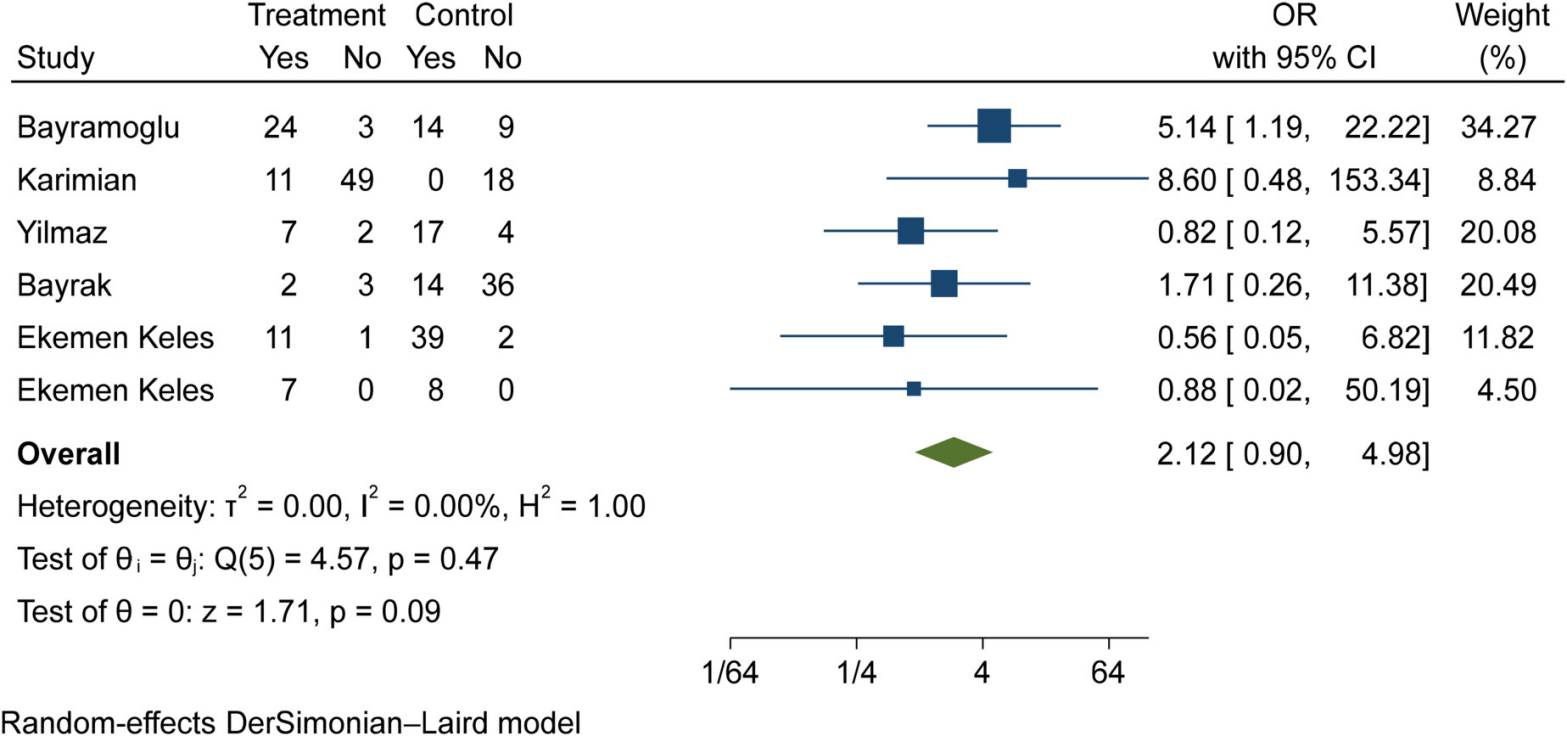
Comparison of vitamin D deficiency among children with moderate COVID-19 compared to the children with mild COVID-19.

The comparison of vitamin D deficiency in children with moderate COVID-19 compared to children with mild COVID-19 was investigated in six primary studies. In three of these studies, the odds ratio of contracting moderate COVID-19 in children with vitamin D deficiency [ranged from 1.71 in Bayrak's study (3) to 8.59 in Karimian's study (27)] was higher than in mild COVID-19 group. Although the observed differences were significant only in the study of Bayramoglu et al. (25). Also, the study of Ekemen Keles (28) has been excluded from the analysis. By combining the results of the preliminary study, the odds ratio of contracting moderate COVID-19 in children with vitamin D deficiency (OR: 2.12, 95% CI: 0.90, 4.98) was 2.12 times that of children with mild COVID-19, which is not statistically significant. It should be noted that the heterogeneity between the studies was not significant (*I*-square: 0%, *Q*: 4.57, *P*-value: 0.47) (Figure 7).

**Figure 8:**
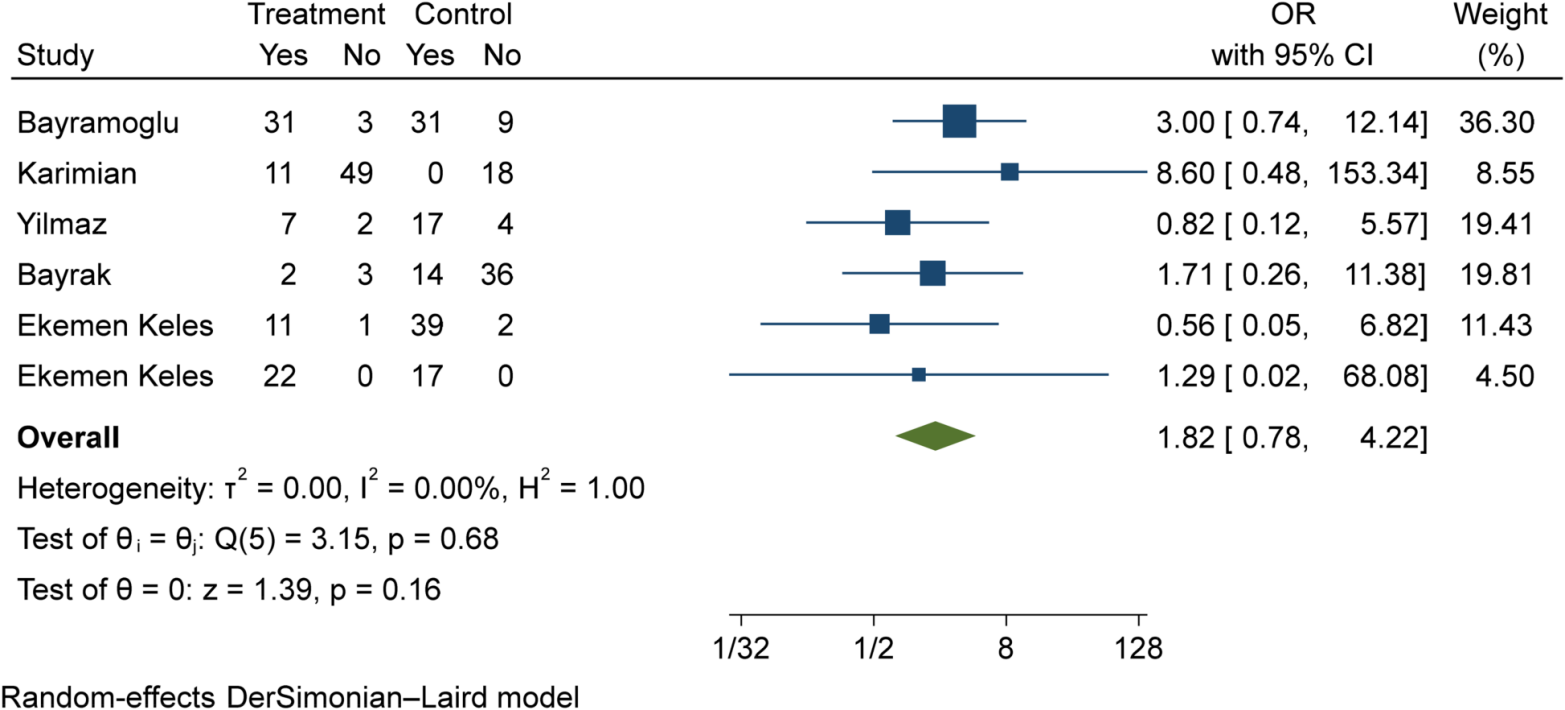
Comparison of vitamin D disorder among children with moderate COVID-19 compared to the children with mild COVID-19.

The comparison of vitamin D disorder in children with moderate COVID-19 compared to children with mild COVID-19 was investigated in six primary studies. In three of these studies, the odds ratio of moderate COVID-19 in children with vitamin D disorder was higher [ranging from 1.71 in Bayrak's study (3) to 8.59 in Karimian's study (27)] than in children with mild COVID-19. There were no statistically significant differences between the studies. Also, the study of Ekemen Keles (28) has been excluded from the analysis. By combining the results of the preliminary study, the odds ratio of contracting moderate COVID-19 in children with vitamin D disorder (OR: 1.82, 95% CI: 0.78, 4.22) was 1.82 times that of children with mild COVID-19, which is not statistically significant. It should be noted that the heterogeneity between the studies was not significant (*I*-square: 0%, *Q*: 3.15, *P*-value: 0.68) (Figure 8).

**Figure 9:**
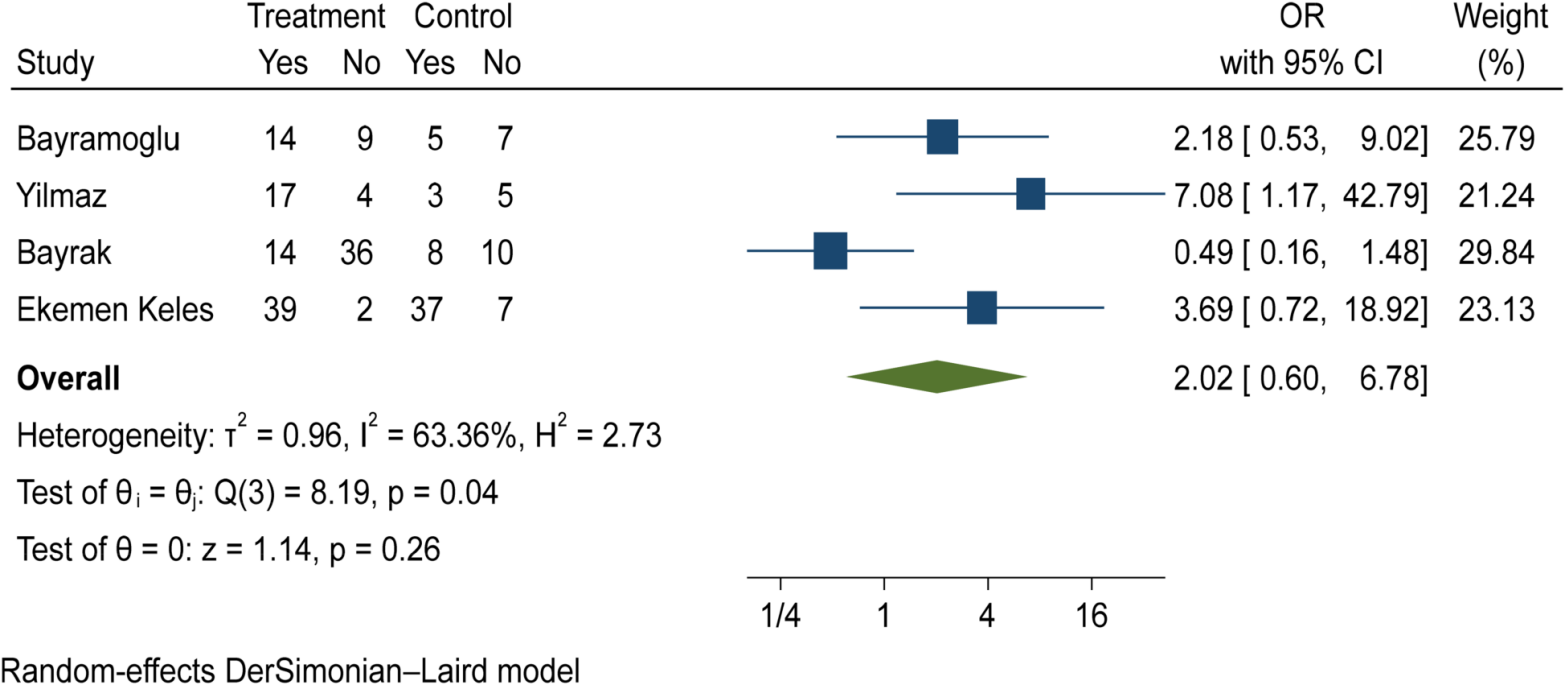
Comparison of vitamin D deficiency among children with mild COVID-19 compared to asymptomatic children with COVID-19.

The comparison of vitamin D deficiency in children with mild COVID-19 compared to asymptomatic children with COVID-19 was investigated in four primary studies. In three studies, the odds ratio of mild COVID-19 in children with vitamin D deficiency [ranged from 2.18 in Bayramoglu's study (25) to 7.08 in Yilmaz's study (26)] was higher than in asymptomatic children with COVID-19. Although the observed differences were significant only in the study of Yilmaz et al. (26). By combining the results of these studies, the odds ratio of contracting mild COVID-19 in children with vitamin D deficiency (OR: 2.02, 95% CI: 0.60, 6.78) was 2.02 times that of asymptomatic children with COVID-19, which is not statistically significant. It should be noted that the heterogeneity between studies has been significant (*I*-square: 63.36%, *Q*: 8.19, *P*-value: 0.04) (Figure 9).

**Figure 10:**
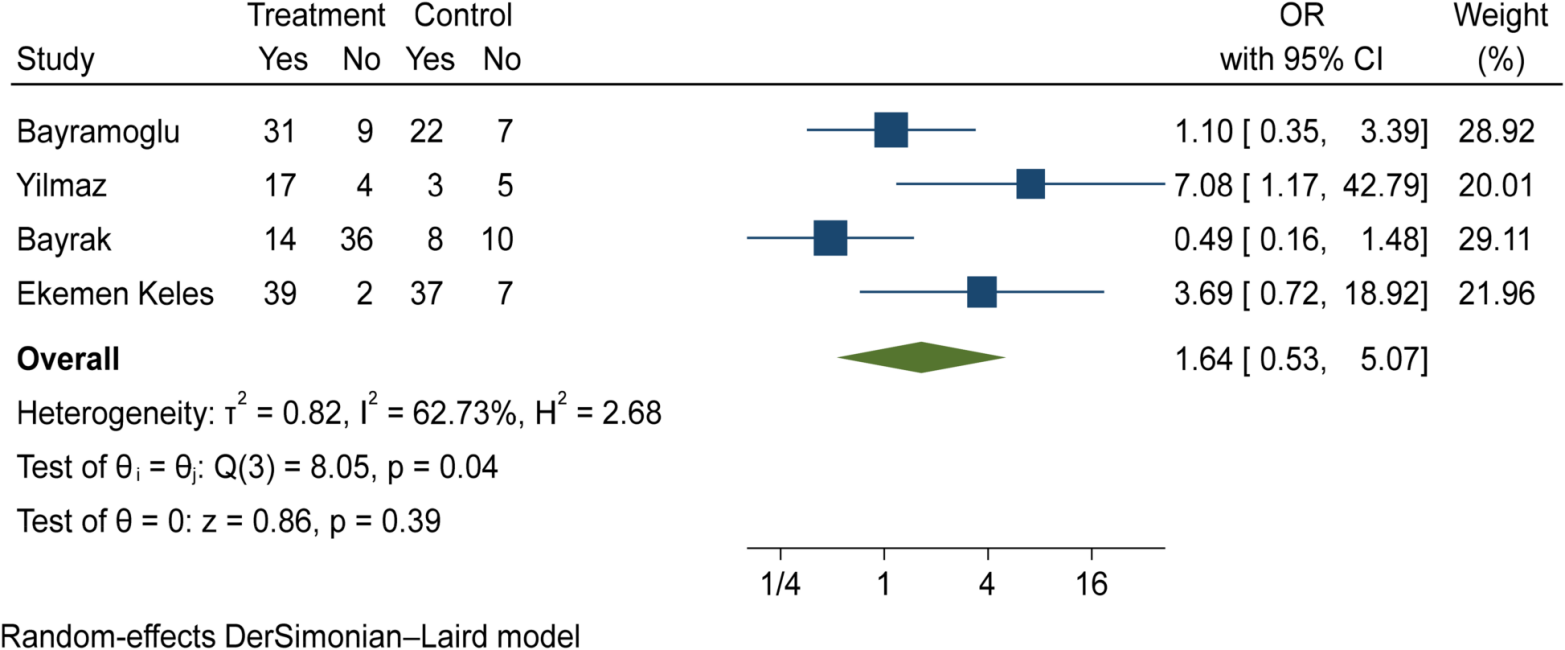
Comparison of vitamin D disorder among children with mild COVID-19 compared to asymptomatic children with COVID-19.

The comparison of vitamin D disorder between children with mild COVID-19 compared to asymptomatic children with COVID-19 was investigated in four primary studies. In three of these studies, the odds ratio of mild COVID-19 among children with vitamin D disorder was higher [ranging from 1.09 in Bayramoglu's study (25) to 7.08 in Yilmaz's study (26)] than in asymptomatic children with COVID-19. Although the observed differences were significant only in the study of Yilmaz et al. (26). By combining the results of these four primary studies, the odds ratio of mild COVID-19 in children with vitamin D disorder (OR: 1.64, 95% CI: 0.53, 5.07) was 1.64 times that of asymptomatic children with COVID-19, which is not statistically significant. It should be noted that the heterogeneity between studies has been significant (*I*-square: 62.73%, *Q*: 8.05, *P*-value: 0.04) (Figure 10).

The comparison of vitamin D disorder between children with severe COVID-19 and those with mild COVID-19 has been extracted from three primary studies. Ekemen Keles' study was not investigated because all children with severe COVID-19 (18 out of 18 people) and mild COVID-19 (17 out of 17 people) were in the vitamin D disorder group. In two studies [Karimian et al.'s study (27) and Yilmaz et al.'s study (26)], the odds ratio of severe COVID-19 among children with vitamin D deficiency was higher than among children with mild COVID-19. The odds ratio in Karimian et al. (27) and Yilmaz's studies (26) were 28.12 and 1.29 respectively and the differences observed in Karimian et al.'s (27) study were statistically significant. It should be noted that because the target group of this study was children, the incidence of severe COVID-19 was low. Therefore, the evidence of vitamin D disorder was not high.

## Discussion

Our study evaluated the status of vitamin D between children with COVID-19 and healthy groups. The SMD of vitamin D in the COVID-19 children compared to the control group was estimated to be −0.88 (98% CI: −1.24, −0.51), which was statistically significant. Also, the odds ratio of moderate COVID-19 among children with vitamin D disorder was higher than in asymptomatic children with COVID-19.

**Figure 11:**
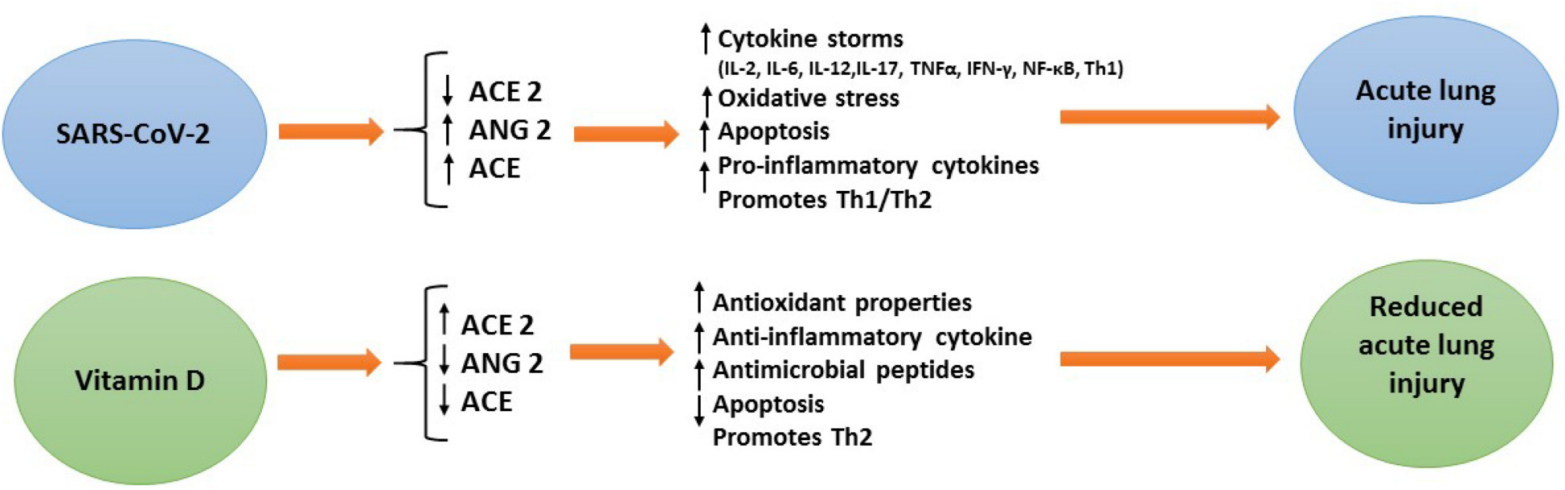
Protective role of vitamin D status against COVID-19.

Vitamin D deficiency is emerging as a global public health problem worldwide. In some countries, vitamin D supplementation has been proposed as a preventive measure for respiratory tract infections (RTIs) in children (26, 29). It has been shown that 1,25-OH2-vitamin D inhibits cellular infection in human nasal epithelial cells and can fight SARS-CoV-2 infections (30). Vitamin D has numerous effects in modulating the immune response. The expression of the vitamin D receptor (VDR) in several immune cells such as dendritic cells, antigen-presenting cells, neutrophils, B cells and T cells, regulates both innate and adaptive immunity (31). It has been reported that patients with COVID-19 reduce plasma cells, pro-inflammatory cytokines [including IL-6, IL-10, granulocyte-colony stimulating factor, macrophage inflammatory protein, tumor necrosis factor-α (TNF-α)] (32), and vitamin D has been shown to increase macrophages' response, anti-inflammatory cytokine production, and activating Toll-like receptors and thus suppress the cytokine storm and pathogenic inflammation during COVID-19 infection (33, 34) (Figure 11).

In addition, it has been reported that the receptor-binding domain (RBD) of the SARS-CoV-2 spike protein enters host cells after it binds to angiotensin-converting enzyme 2 (ACE2) receptors (35). Vitamin D can balance the inflammatory response by increasing the expression and concentration of ACE2, which has anti-inflammatory properties and is capable of trapping and inactivating virus particles. Genetic variations in the vitamin D receptor (VDR) gene or ACE2 may also affect COVID-19 disease outcomes (36). A study (37) suggested that sufficient vitamin D boosts the innate immune system and improves the adaptive immune response so reduces the risk of acute respiratory tract infection (such as respiratory syncytial virus, tuberculosis, and influenza) (12, 38). Yilmaz and Şen (26) investigated the correlation between vitamin D and COVID-19 in a pediatric age group. They also showed that vitamin D was significantly lower in COVID-19 patients than in the controls. Martineau et al. (39) reported that 25(OH)-D vitamin is generally protective against acute respiratory tract infection. In the study of Li et al. (40) with 1,582 children, the group with community-acquired pneumonia was compared with the control group, and the low 25(OH)-D vitamin was found to be lower in the group with commu­nity-acquired pneumonia. In addition, in a case-control study conducted by Velarde López et al. (41) 25(OH)-D vitamin deficiency was found to be significantly low in children with lower respiratory tract infections. Previous studies in critically ill children have shown an association between severe disease and vitamin D deficiency (26, 27). Moreover, a recent study reported that a single dose of vitamin D given to pediatric patients with vitamin D deficiency could reduce the incidence of septic shock in specific patient populations (42) Akoğlu et al. (43) also found that the 25-hydroxyvitamin D3 (vitamin D3) was significantly lower in the moderately severe disease group than the mild disease group (*P* = 0.044).

A study evaluating Indian children found that acute lower respiratory infection was significantly higher among children with vitamin D deficiency in a six-month follow-up time (44). Banajeh investigated 79 cases between 2 and 59 months and found that vitamin D deficiency was associated with reduced circulating neutrophils and hypoxemia (45). Akoğlu et al. (43) reported that patients with moderate COVID-19 severity had lower 25(OH) D as compared to the mild disease group. An epidemiological study reported that 25(OH) D < 20 ng/ml almost doubled the risk for SARS-CoV-2 infection and hospitalization (46). Merzon et al. defined in their study that <30 ng/ml was a risk factor for infection and hospitalization, independent of demographic characteristics and previous medical conditions (46). In the study conducted by Carpagnano et al. (47), mortality was shown to be 50% higher in patients hospitalized with COVID-19 infection with acute respiratory failure in patients with 25(OH)-D < 10 ng/ml. During the COVID-19 pandemic (except for the Omicron peak), children were less affected by the severity of COVID-19. Combining the results of these studies, the effect size of the relationship between vitamin D and COVID-19 severity in children is significant. The standardized mean difference (SMD) vitamin D level in children with COVID-19 was significantly 0.88 units lower than the control group. Also, the odds ratio of moderate COVID-19 in children with vitamin D deficiency was significantly 3.58 times higher than in asymptomatic children with COVID-19.

## Limitation

This study has some limitations:

At first, the relationship of zinc and vitamin D to disease incidence, contribution to treatment and clinical severity was not examined, which can be the subject of a new approach. Second, weight and some clinical symptoms such as abdominal pain, pharyngeal pain, loss of taste, and myalgia cannot be evaluated in all groups. Third, most studies were from Turkey and Egypt. Therefore, this limitation should be explained in the interpretation of the results.

Another limitation of the present study is that there is a possibility that vitamin D in the patients with COVID-19 was low before contracting the disease, and it is not possible to prove this in this meta-analysis due to the lack of access to vitamin D data.

## Data Availability

The original contributions presented in the study are included in the article/supplementary material, further inquiries can be directed to the corresponding author/s.
